# National Association of General Practitioners

**Published:** 1845-04

**Authors:** 


### THE NATIONAL ASSOCIATION OF GENERAL PRACTITIONERS IN MEDICINE, SURGERY, AND MIDWIFERY.

In the article in our last Number, in which we examined, at such length, the new Medical Bill in all its bearings, we stated, after much consideration, "that we had come to the conclusion that it would be for the benefit of the profession generally, that, in addition to the existing medical corporations, a third should be established for the class of general practitioners." The consideration of the same facts, and the same process of reasoning, that led us to this conclusion, seem to have impressed the minds of the great body of general practitioners in the same manner; and the result has been the formation of an Association to carry into effect this important object. The association has been carefully organized, and its operations have been throughout conducted in that spirit of mingled caution and activity, which are at once indicative of sound sense in the present, and prophetic of sure success in the future. The Association has already enrolled about 4000 members, and is proceeding to increase its numbers at the rate of 30 to 40 daily. Indeed, it may be said that the whole of the general practitioners have taken up the question, and it can no longer be doubtful that the object they have in view must be attained, provided they continue to act with the energy and discretion they have hitherto displayed.

The following extract from the Report of the Provisional Committee of the Association, presented to the general meeting held on the 14th March, contains an exposition of the present views of the members as to the nature of the institution they are seeking to establish, as well as a summary of the principal changes which appear to them requisite in the new Medical Bill; it also exemplifies the prudent and conciliatory spirit in the management, which we have been commending.

---

"Since the promulgation of the heads of Charter, the committee have had a very extensive correspondence on the subject. The result is, that they are prepared to recommend some modification in the original suggestions. Every advice from the country has been in favour of voting papers, either available personally or by proxy, and the committee most cordially agree in recommending the meeting to adopt that plan. Every advice from the country has been in favour of a ten years' instead of a five years' franchise. The committee are at the same time aware, that there is a numerous class in the metropolis who desire that every qualified man should have a vote. Having well considered all the bearings of this question, they are disposed to recommend the five years' franchise as originally suggested by themselves. The proposals for a Charter by the committee, at Manchester, embrace many details with which your provisional committee consider it would be inconvenient to encumber the first suggestions, and in particular, having in view a distinction between those practitioners who combine the character of tradesmen with their professional calling, the Manchester committee propose a distinction of Fellows and Members in the New College: most cordially concurring in the object, your provisional committee would enquire whether the best mode of attaining that, and many other most desirable objects, will not be through the medium of a well considered, and duly sanctioned code of bye-laws?

There are some minor changes made in the "Heads of Charter," and the committee submit the following as the form of incorporation which would be best received, and most calculated to promote the welfare of general practitioners, and the public good.

'*College of General Practitioners in Medicine, Surgery, and Midwifery;' under an appropriate Title, with a Common Seal, and power to sue and be sued, and to hold Lands and Tenements not exceeding £5000 per annum.*

MEMBERS TO BE INCORPORATED.

*In the first instance.* 1. Every gentleman who was in actual practice previous to the 1st of August, in the year 1845. 2. Every Licentiate of the Apothecaries' Company. 3. Every Member of the Royal Colleges of Surgeons in England, Ireland, or Scotland:—Every Doctor or Bachelor in Medicine, of any University of the United Kingdom. 4. Every Fellow or Licentiate of any College of Physicians of the United Kingdom who shall have been respectively in actual practice as a General Practitioner in England or Wales at the period of the granting of the Charter, and who shall be enrolled a Member within twelve months from the date of the Charter.

*Subsequently.* Such persons only as shall have been duly examined and certified and shall have brought themselves within the scope of the Charter and the Bye-laws.

GOVERNING BODY.

*One President,* to be elected by the Council triennially and to be eligible for re-election.

*Three Vice-Presidents,* to be elected by the Council from their own body by ballot, one to go out every year, and not to be eligible for re-election for a year.

*A Council,* to be composed of Forty-eight or Sixty Members, one half Practitioners resident within ten miles of the Royal Exchange, the other half Country Practitioners resident beyond that distance. No Member to be eligible as a Member of Council under Fifteen Years standing as a General Practitioner.

The first President, Vice-Presidents, and Council to be nominated in the Charter, and to hold their offices for three years.

All future Members of Council to be elected by Members of the College whose Diploma or License shall bear date not less than Five Years previous to the day of Election.

After the expiration of the first Three Years, one third of each branch of the Council to go out of office annually, and the vacancies to be filled up in equal proportions from the same branches, but the retiring Members not to be eligible for re-election for a year.

The Election to be by voting papers and decided by a majority of votes.

The Council to have the power of directing the Course of Study for the future Members of the College.

The Council to have the power of framing Bye-laws, Rules, and Ordinances, with power of administering oaths for the regulation of the affairs of the College, subject to the revision of the Law Officers of the Crown.

The Council to have the power of appointing and dismissing all Stipendiary Officers.

The Fees to be payable upon the Examination of Candidates as well as the Annual Contributions of Members, to be defined in the Charter.

COURT OF EXAMINERS.

The Council to elect annually a Court of Eighteen Examiners.

No Member of Council to be eligible for that Court.

No Member of the College to be eligible as an Examiner under Ten Years' standing from the date of his Diploma or License.

The President or one of the Vice-Presidents to preside at every Meeting of the Court of Examiners, but not to have any Vote.

Diplomas to practise as General Practitioners to be granted under the College Seal.

The chief modifications which the Bill would require to give effect to this Charter are:

I. The total repeal of the Apothecaries' Act, as originally intended by the Minister.

II. To give the Council of the new College, by law, Representatives in the Council of Health—as the Minister promised for his own part in his speech on the introduction of the Bill.

III. To register under the Bill the Members of the new College of General Practitioners in the place of Licentiates in Medicine and Surgery.

The further changes in the principles of the bill which your provisional committee deem necessary to secure the welfare and true respectability of the class of general practitioners, and, indeed, of the whole profession, are—

I. A more efficient penal clause against unlicensed practitioners.

II. A withdrawal from the Council of Health of the power of designating what class in the profession shall be eligible to public offices, leaving the power in the hands of the public as heretofore of choosing their medical attendants, whether for public or private practice, from any class of qualified medical practitioners.—

With some important changes in the construction of certain clauses of the bill, easily introduced in committee, as—

I. That no person be admitted to examination under 22 years of age.

II. That no person be admitted to examination without testimonials of having devoted at least five years to medical study.

III. That testimonials of a similar period of study be required from the class of inceptor candidates.

IV. That the special examination in midwifery be open to none but registered practitioners of one class or other.

In fact every modification of the Bill should have a direct tendency to elevate the character, and increase the usefulness of all classes of medical practitioners alike.

The Committee request the meeting most particularly to observe, that the Heads of Charter are brought before them strictly as "*suggestions*," and as such they are herein recorded; so that, should it be thought proper to adopt this Report, the association will not be precluded from discussing and amending the principles and the details embraced.

No efforts have been spared, in the endeavour to reconcile conflicting opinions, and to frame such an outline as will give general satisfaction. Many individuals who have agreed to these suggestions, have more or less modified their original views, for the purpose of promoting unanimity; and without the slightest desire to interfere with perfect freedom of discussion the Committee beg most urgently to impress upon their audience, that dissension will be fatal to the attainment of our just rights, while unanimity of opinion, and cordial co-operation, with a definite object, having for its basis the public good, will most certainly, sooner or later, be crowned with success."

---

While approving generally of the above sketch of the projected Charter, there are several things in it which we think might be modified with advantage; and there is one point of vital importance, respecting which we differ entirely with the Association. Our nearly exhausted limits will permit us merely to refer to the subject at present; but we earnestly call the attention of the Committee to it, as a matter of the greatest importance to the whole medical profession: we refer to the EXAMINATION BOARD, or COURT OF EXAMINERS.

We remain of the opinion, so strongly expressed in our last Number, that there should be only ONE Examination Board for testing qualifications for practice, and that every one entering the profession, to whatever class he might belong, should be obliged to pass through it; the COLLEGES being left to establish whatever examinations they chose for the *admission of their own members.* We think that the institution of a distinct Examination Board for general practitioners would tend not only to separate them still farther from their brethren of the other classes of the profession, but would have the effect of lowering, instead of elevating them as one of the constituent bodies of the profession. We admit that they are fully justified in objecting to the examination Board proposed in the Bill; but we think they should exert their influence, rather to obtain one general Board for all classes, than a special Board for their own. We sincerely desire to see the new College established; but we wish its establishment to be a new bond of union to the profession, not a means of further disunion. We wish it to have all the rights and privileges of an independent College; but we wish it to have no more. Our desire is to see the exorbitant claims and pretensions of the existing Colleges reduced within legitimate limits, not to see the new College claiming a participation in powers which are inconsistent with the general good of the profession.

As the readiest mode of stating our general views respecting the organization of the profession, we reprint the following paragraph from our last Number; and we venture to assert that the plan sketched by us, of which this is an outline, contains the elements of a system of organization, calculated to produce greater unity and harmony, and more general good to the profession than can result under the Medical Bill, even when modified according to the provisions of the proposed Charter.

"I. A general Governing Board (The Council of Health and Medical Education), having the exclusive regulation of the education, mode of examination, registration, and licensing of medical practitioners of every class.

"II. An Examination Board, appointed by the Council of Health and unconnected with the Colleges, for testing the qualifications of candidates for the license to practise. No one to be permitted to enter the profession without being examined by this Board, and obtaining from it Letters testimonial to the Council of Health, authorizing the granting of the License to practise as a Licentiate of Medicine and Surgery; and no one, not possessing this License, to have any legal right to practise in any department of the profession.

"III. A College of Medicine and Surgery for the [voluntary] incorporation of Licentiates of Medicine and Surgery, to be instituted by Royal charter under the name of 'The Royal College of Medicine and Surgery,' with power to frame laws for the admission of its members (Members and Fellows), for the government of its affairs, electing officers, &c. &c.

"IV. A College of Surgery (The Royal College of Surgeons of England), with power to frame laws for the admission of its members (Members and Fellows), &c. &c.

"V. A College of Medicine (The Royal College of Physicians of England), with power to frame laws for the admission of its members (Associates and Fellows), &c. &c."

